# Paleochannel and beach-bar palimpsest topography as initial substrate for coralligenous buildups offshore Venice, Italy

**DOI:** 10.1038/s41598-017-01483-z

**Published:** 2017-05-02

**Authors:** Luigi Tosi, Massimo Zecchin, Fulvio Franchi, Andrea Bergamasco, Cristina Da Lio, Luca Baradello, Claudio Mazzoli, Paolo Montagna, Marco Taviani, Davide Tagliapietra, Eleonora Carol, Gianluca Franceschini, Otello Giovanardi, Sandra Donnici

**Affiliations:** 1 Institute of Marine Sciences, National Research Council, Arsenale - Tesa 104, Castello 2737/F, 30122 Venezia, Italy; 2 National Institute of Oceanography and Experimental Geophysics, Borgo Grotta Gigante, 42/c, 34010 Sgonico, Trieste, Italy; 3 Botswana International University of Science and Technology, Private Bag 16, Plot, 10071 Palapye Botswana; 4 Institute of Marine Sciences, National Research Council, Via Gobetti 101, 40129 Bologna, Italy; 5 Department of Geosciences, University of Padova, Via Gradenigo 6, 35131 Padova, Italy; 6 Laboratoire des Sciences du Climat et de l’Environnement LSCE/IPSL, CEACNRS-UVSQ, Université Paris-Saclay, Avenue de la Terrasse, Gif-sur-Yvette, Île-de-France 91198 France; 7 Biology Department, Woods Hole Oceanographic Institution, 266 Woods Hole Rd, Woods Hole, Ma 02543 USA; 8 Stazione Zoologica Anton Dohrn, Villa Comunale, 80121 Napoli Italy; 9 Centro de Investigaciones Geologicas, Consejo Nacional de Investigaciones Científicas y Tecnicas, Diagonal 113 N275, B1904DPK La Plata, Argentina; 10 Italian National Institute for Environmental Protection and Research, Loc. Brondolo, 30015 Chioggia, Venezia Italy

## Abstract

We provide a model for the genesis of Holocene coralligenous buildups occurring in the northwestern Adriatic Sea offshore Venice at 17–24 m depth. High-resolution geophysical surveys and underwater SCUBA diving reconnaissance revealed meandering shaped morphologies underneath bio-concretionned rocky buildups. These morphologies are inferred to have been inherited from Pleistocene fluvial systems reactivated as tidal channels during the post- Last Glacial Maximum transgression, when the study area was a lagoon protected by a sandy barrier. The lithification of the sandy fossil channel-levee systems is estimated to have occurred at ca. 7 cal. ka BP, likely due to the interaction between marine and less saline fluids related to onshore freshwater discharge at sea through a sealed water-table. The carbonate-cemented sandy layers served as nucleus for subsequent coralligenous buildups growth.

## Introduction

The northwestern Adriatic Sea bottom consists of Holocene siliciclastic marine sediments grading into Pleistocene continental deposits in the offshore, at about 15–25 m water depth e.g. refs 1–5. The occurrence of localized bio-concretionned rocky buildups, widespread along the northwestern Adriatic Sea inner shelf between 10 and 40 m depths, is anecdotally known since the 18^th^ century^6^. The rocky buildups rise up to 3–4 m above the siliciclastic sea floor and their exact locations have been identified through time by fishermen attracted by their fishing value and also because they represent a potential threat for bottom trawling. These rocky buildups, according to the traditions of the local fishermen, are known under various dialectal names, e.g., *tegnùe*, *trezze*, *pressure*, *lastrure*, *grèbeni*.

These rocky buildups have a patchy distribution and a variety of benthic calcareous constituents contribute to their growth, including bryozoans, mollusks, serpulid polychaetes, scleractinians and calcareous algae, the latter acting as the main bio-concretionning organisms [ref. 7 and references therein].

The northwestern Adriatic bio-concretionned rocky buildups show a significant similarity with the coralligenous habitat and they may fall within the definition of coralligenous biocenosis [ref. 8 and references therein]. However, their composition is different from typical Mediterranean coralligenous assemblages^9^ and recently they have been categorized as coralligenous outcrops subdivisible into three main habitats along an onshore-offshore gradient^10^.

Their ecological role is rather significant since providing a number of ecosystemic services, from fisheries to recreational e.g. ref. 11. For instance, they offer shelter, reproduction and nursery ground to fish and invertebrate species, including some under stress due to severe fishing pressure e.g. ref. 10, what justifies their protection by European and regional laws, i.e. European Marine Protected Areas, Biological Protection Zones. In addition, these northern Adriatic peculiar bio-concretionned buildups are underwater sites deserving to be preserved for their unique geo-biological legacy.

The obvious richness in marine fauna and flora has resulted in a number of studies mainly devoted to specific or general aspects of the ecology [e.g. ref. 10 and references therein], while less has been done to unravel the peculiarity of the siliciclastic substratum that allowed the inception and growth of the reef-building organisms. Recognizing the initial steps of bio-concretionned rocky buildups developing in a loose silicilastic sedimentary context has been always hampered by the difficulty of getting samples of diagnostic value, especially for the recurrent conditions of poor visibility due to the high water turbidity encountered by divers. Marine geologists tried in the past to unravel the prime process at the base of sediment lithification originating these rocky buildups. Starting from the ‘60 s the most accredited hypothesis describes these features as representing Holocene beachrocks formed at a very early stage of the post-glacial transgression e.g. ref. 12. Further investigation highlighted marked erosion along the northern Adriatic shelf suggesting that the bio-concretions likely grew on structural relicts of exhumed sedimentary horizons e.g. ref. 13. During the last two decades, additional data and observations supported the idea that the rocky buildups, in the northwestern Adriatic Sea, are linked to calcium carbonate precipitation triggered by anaerobic oxidation of methane e.g. ref. 14. Recent investigations provided evidence that at least a part of the northern Adriatic bio-concretionned buildups are located in the nearby of gas seep occurrences e.g. refs 15 and 16.

Previous geological studies have clearly shown that the northwestern Adriatic Sea rocky buildups are Geosites of worldwide scientific interest and understanding their genesis may lead to improve the knowledge on the paleoclimate and geological evolution of the Adriatic Sea. The existing genetic models for the rocky buildups in this area lack a description of the paleo-environment evolution that leads to the early diagenesis of the substrate and subsequently the growth of the coralligenous.

Although the aforementioned studies have provided data supporting various hypotheses on the cementation processes of the inner shelf sediments of the north-western Adriatic, to date many questions on the formation of the hardened sediments that provided the first substrate to the coralligenous habitats are still open. For instance, when did the sediments at the base of the bio-concretions consolidate? What particular environmental conditions existed at the time of cementation? Why do the bio-concretionned buildups show different morphologies and why do some of them occur along quite continuous meandering outcrops between 15 and 23 m water depth? These questions can be addressed by combining geophysical, petrographic and geochemical analyses to assess the genetic model of coralligenous buildups within the framework of the geological evolution of the north-western Adriatic Shelf.

**Figure 1:**
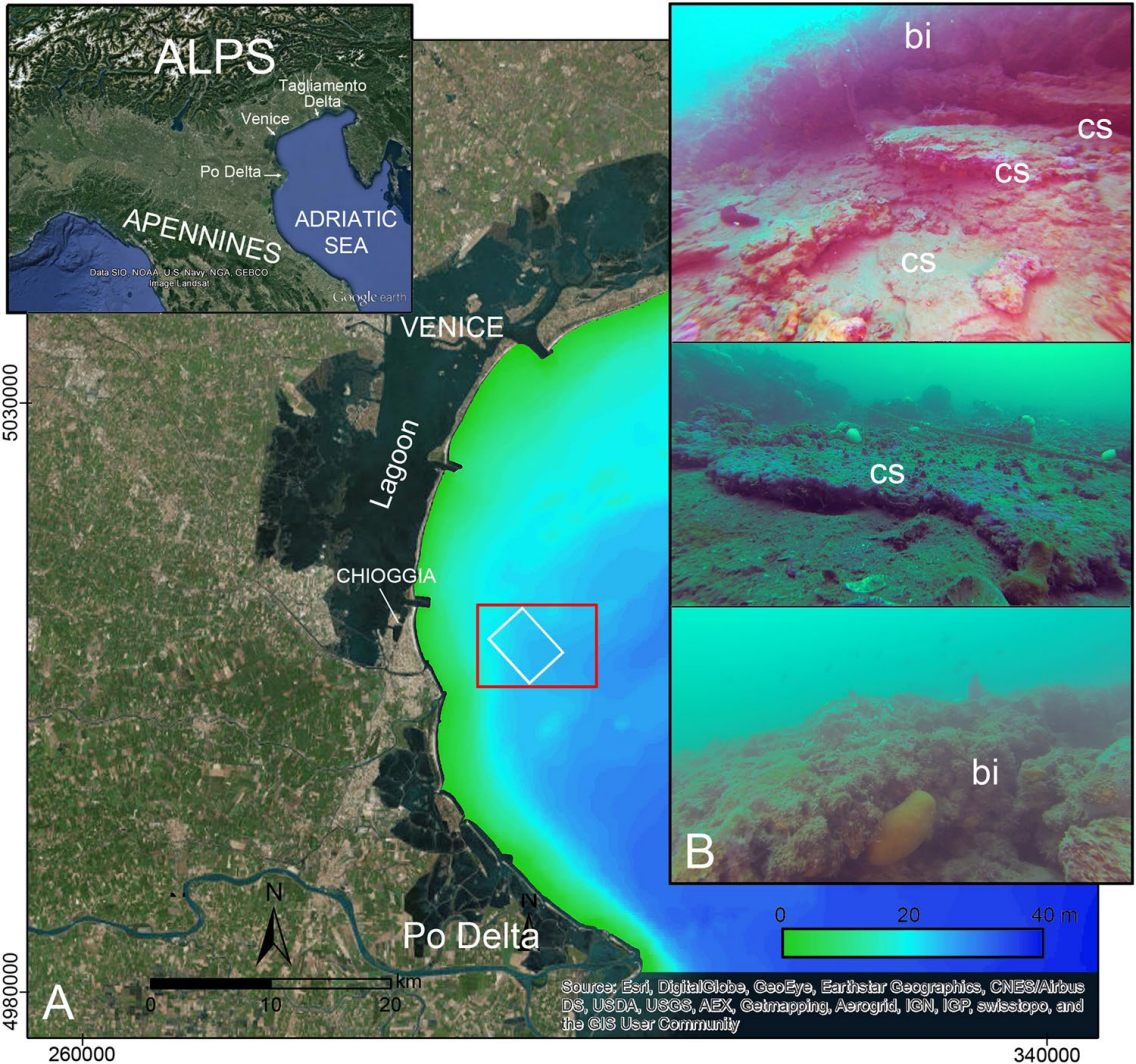
(**A**) Location of the study area (red rectangle) in the northern Po River Delta and Venice coastland (UTM 33N WGS84). White rectangle shows the area of the shaded relief map of Fig. 2. Base maps are Google Earth and Esri, DigitalGlobe, GeoEye, Earthstar Geographics, CNES/Airbus DS, USDA, USGS, AEX, Getmapping, Aerogrid, IGN, IGP, swisstopo, and the GIS User Community; bathymetric data are from EMODnet Bathymetry Consortium, http://doi.org/10.12770/c7b53704-999d-4721-b1a3-04ec60c87238 ^43^. Images are composed in ESRI ArcMAP 9.3. (**B**) Underwater photographs of rocky buildups (tegnùe): cemented sand layers (cs) at the base of the bioconstructions (bi) are shown in (**B**).

This work aims at providing a genetic model of the rocky buildups 5–8 km offshore Chioggia, South of Venice (Fig. 1A). This is a Biological Protection Zone since 2002, where the sea bottom is shaped by bio-concretionned formations depicting peculiar and unique morphologies^17^. The bio-concretionned buildups occurring in the Chioggia offshore are known as tegnùe and the biological concretions represent a shallow water coralligenous habitat [e.g. ref. 7 and references therein] (Fig. 1B,C,D). Geophysical data have shown the surface and sub-surface morpho-architecture of the bio-concretionned rocky buildups creating the framework for underwater SCUBA divers’ direct geomorphological observation and accurate sampling. The analysis of sediment and rock samples provided information on the cementation process, paleo-environment depositions and lithification age. These data, integrated with the knowledge of the regional stratigraphic and evolution setting of the study area, allowed us to provide a new genetic model of the *tegnùe*.

## Results

**Figure 2:**
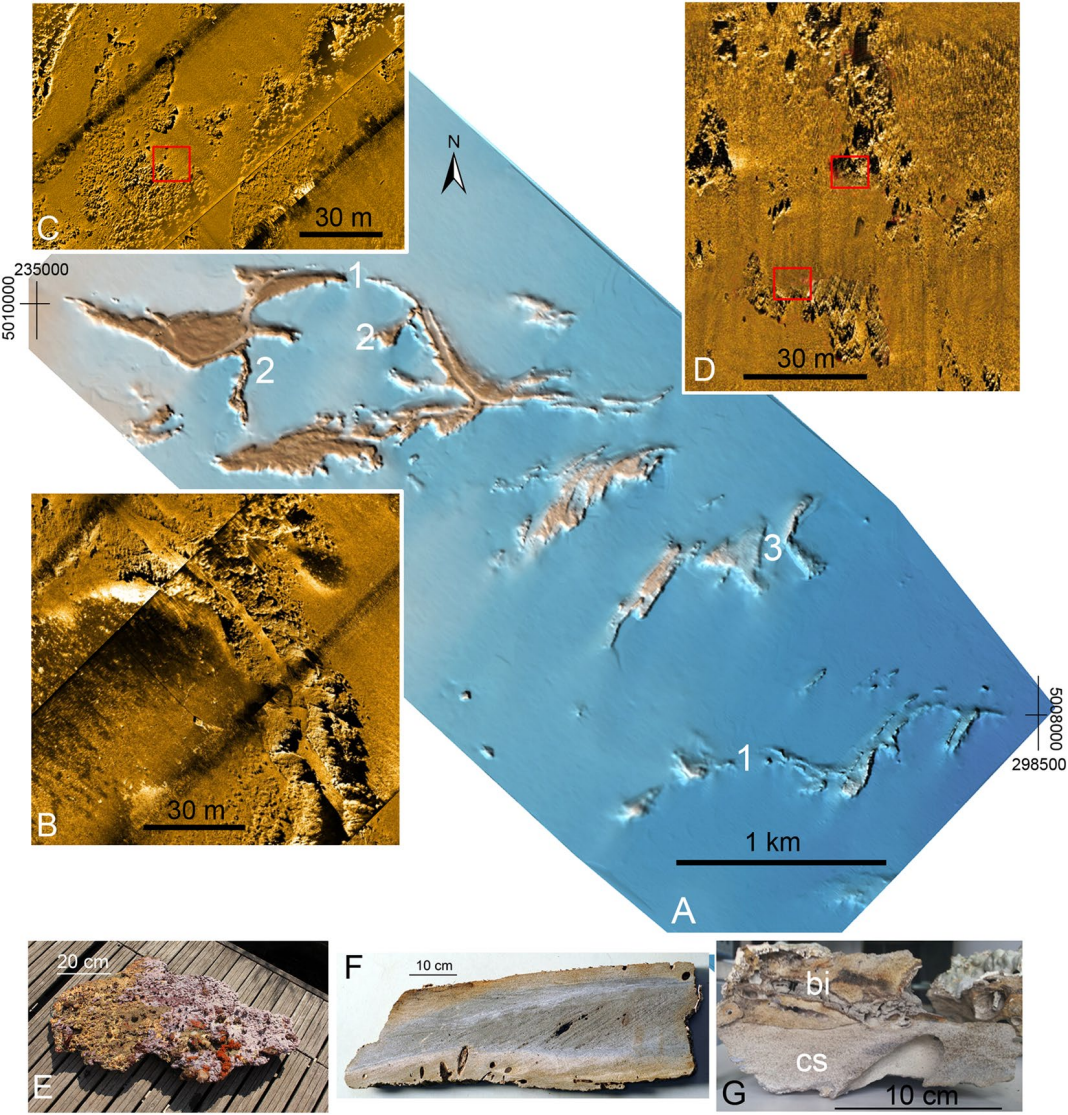
(**A**) Shaded relief map obtained by swath bathymetry (UTM 33N WGS84) (see location in Fig. 1). The water depth are between 17 and 25 m and bio-concretionned rocky buildups rise up to 3–4 m from the sandy sea bottom. Meandering-like horizontal paths (1) fan-like features (2), arc-shaped features (3). (**B**,**C** and **D**) Side Scan Sonar images showing examples of bio-concretionned rocky buildups morphologies. (**E**) top of the cemented shell-rich layer found at the seafloor (sampling location: bottom red square in (**D**). (**F**) Cemented sand sample (sampling location: bottom red square in (**C**). (**G**) Sample of tegnùe highlighting bio-concretions (bi) with the cemented sand layer (cs) at the bottom (sampling location: upper red square in (**C**). Additional images of cemented sand samples are in Supplementary Material Figure S4.

Combined high-resolution swath bathymetry and side scan sonar images reveal extraordinary growth of coralligenous buildups in the *tegnùe* field of Chioggia, at water depths ranging from 17 to 24 m (Fig. 2A,B,C). Bio-concretionned rocky buildups rise up to 3–4 m from the sandy sea bottom and develop along specific winding paths. In particular, in the northern part of the study area, they exhibit hectometer- to kilometer-scale meandering-like horizontal paths, and their growth produces two parallel trails 20 to 50 m apart separated by a trough (Fig. 2A,B). Minor winding ridges, connected to the main meandering structure, locally terminate as fan-like features (Fig. 2A). A less prominent meandering structure, composed of two parallel ridges, is found toward the south of the study area (Fig. 2A). In the central part of the study area, the coralligenous buildups delineate hectometer-scale arc-shaped features, whose concavity is oriented to the SE direction (Fig. 2A).

**Figure 3:**
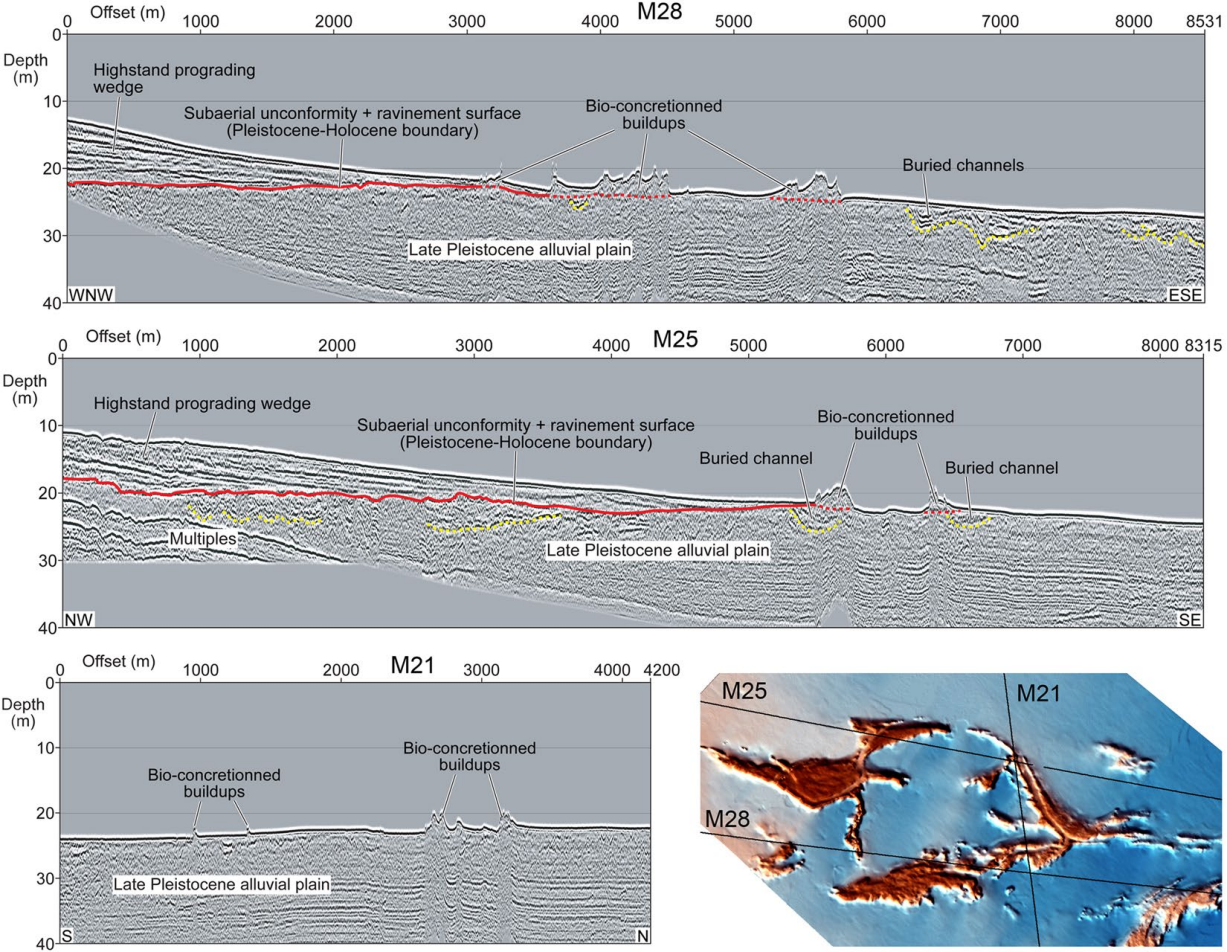
(**A**,**B**,**C**) Examples of High-resolution seismic surveys crossing the tegnùe field. Yellow and red lines highlight major channelized features and the contact between Holocene and Pleistocene seismic units, respectively. The inset shows the seismic line positions superposed to the swath bathymetry shaded relief map. Seismic images obtained by IHS Kingdom® software educational licence (https://www.ihs.com/products/educational-grant.html) and edited with Gimp 2.8.14.1 (https://www.gimp.org).

High-resolution seismic surveys planned on the basis of the morpho-bathymetric data allowed to frame the subsoil architecture (Fig. 3; Supplementary Material Figures S1–S3). This consists of a lower unit, composed of gently seaward-inclined to irregular, low- to high-amplitude reflectors and locally channelized features, overlain by a sedimentary body that wedges-out seaward and is composed of high-amplitude reflectors that dip in the same direction and downlap the top of the lower unit (Fig. 3, Supplementary Material Figures S2 and S3). The contact between the two seismic units is irregular, sub-horizontal and merges with the seafloor in a seaward direction (Fig. 3A,B, Supplementary Material Figures S2 and S3). In the seismic lines the bio-concretionned rocky buildups appear as very irregular features on the seafloor, lying just off the seaward termination of the upper unit, and in some cases buried channel structures are recognizable just below them (Fig. 3A,B,C, Supplementary Material Figure S3).

Underwater scientific SCUBA divers recognized that the coralligenous buildups rest on well cemented sand (Fig. 1B,D, Supplementary Material Figure S4).

**Figure 4:**
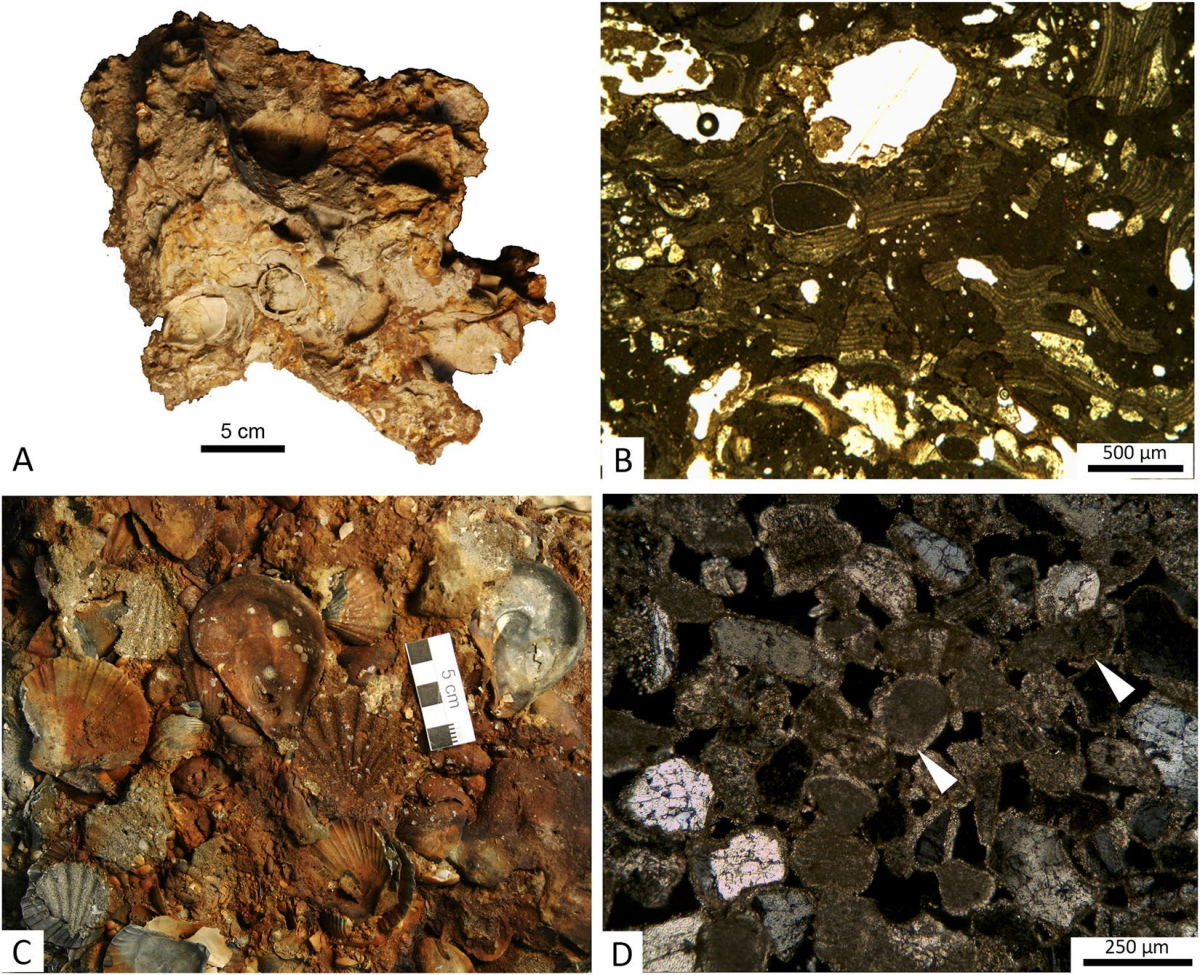
Hand specimens and photomicrographs of the lithotypes described among the *tegnùe* of Chioggia. (**A**) Sample of biofouled bioclastic rock. (**B**) Photomicrograph (transmitted light, plane polarized light) of the bioclastic rock (**A**) showing millimeter-sized clasts of red algae and turbid micrite cements. (**C**) Particular of a slab of shell-rich coarse layer interpreted as the post-LGM transgressive lag (sample MT_01_14). (**D**) Photomicrograph (transmitted light, cross polarized light) of the mixed siliciclastic carbonate sandstones that builds the substrate for the bioconstructions. Arrows point to an isopachous generation of cement.

**Figure 5:**
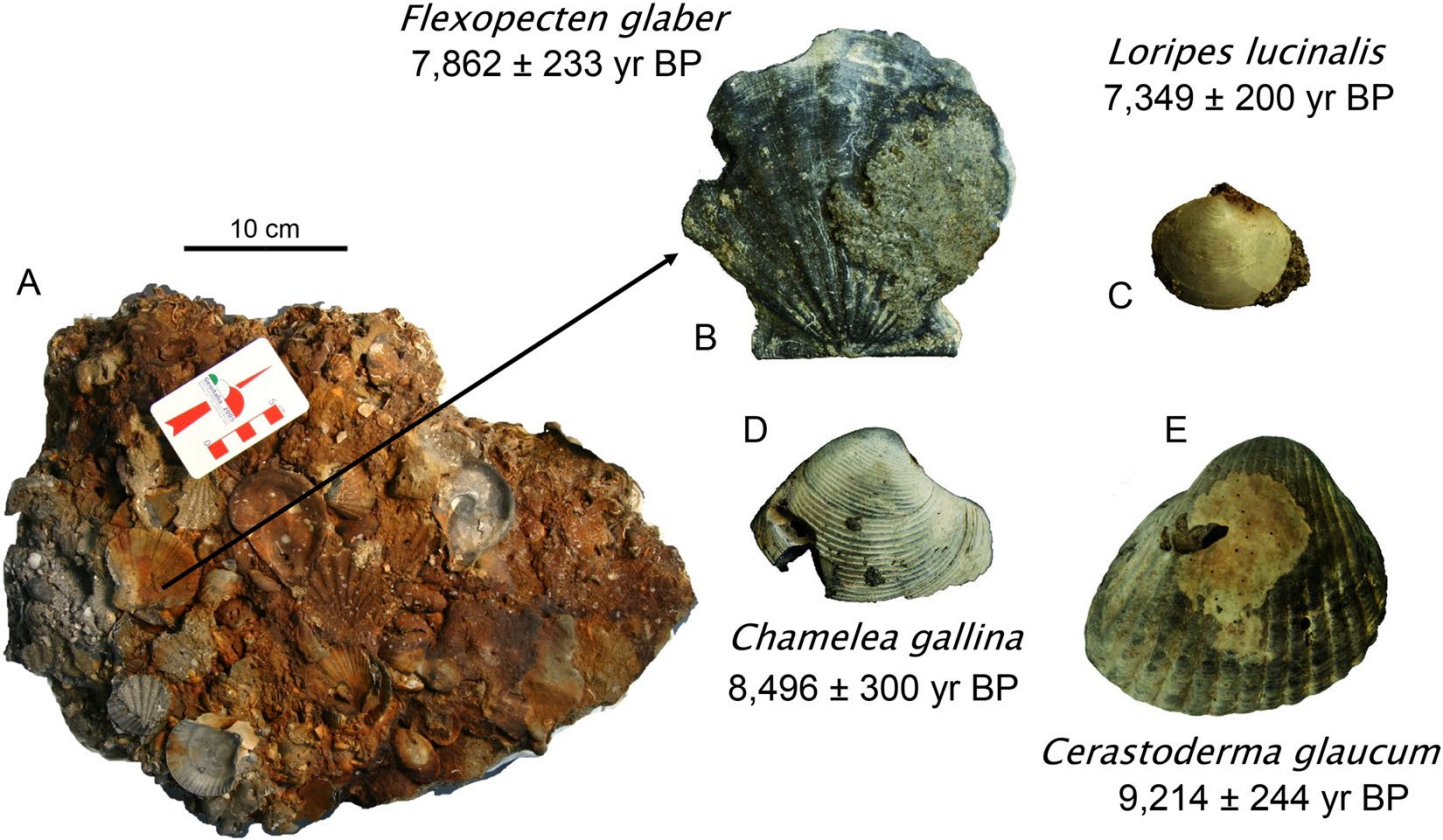
Large mixed siliciclastic carbonate sand slab (MT_01_14) embedding a variety of bioclasts and biosomes from various environments (freshwater, brackish-lagoonal and shallow marine) and AMS-^14^C ages of selected bivalve shells.

In the bio-concretion buildups, the biogenic detritus consisting of serpulids, red algae, bryozoans, and bivalves fills the cavities and the primary voids left by the irregular superposition of the builders, and it undergoes an early cementation (Fig. 4A,B). Visual inspection of the samples of coralligenous collected along a 2 m vertical transect across one of these bio-concretionned rocks revealed that they are mostly made up of millimetric fragments of red algae and bivalve shells cemented by micrite and coated by red algae (Fig. 4B). The rocky substrate of the bio-concretions is composed of weakly-lithified grey sandstone with silicate and carbonate grains (Fig. 4D). Petrographic and diffractometric analyses revealed that most of the terrigenous materials consists of well sorted rounded clasts of micritic limestone, dolomite and angular clasts of quartz with subordinate glauconite (Fig. 4D). Texturally the sandstone is grain supported with a low degree of compaction as shown by the relatively small number of grain-to-grain contacts, the absence of pressure solution, and the high porosity. A low-Mg calcite cement consisting of scalenohedral crystals forming fringes few tens of micrometers thick (Fig. 4D), joins adjacent grains and only partially invades larger pores. Locally the sandstones retain cross-bedding and lack evidences of biofouling (Supplementary Material Figure S5A). A ca. 10–20 cm thick layer composed of shell-rich cemented sand was locally found just at the seafloor (Fig. 2E; Supplementary Material Figure S5B). The shell-rich coarse layer consists of sandstone with abundant biosomes (Figs 4C and 5). This sandstone is petrographically and mineralogically comparable to the sandstone described in Fig. 4D. One key sample provided a much needed control on the timing of the early steps of sediment lithification. Sample MT_01_14 was taken intact to the surface (Fig. 5), and embeds a variety of disarticulated and locally imbricated shell remains, pertaining to a variety of freshwater, brackish and shallow marine habitats sourced from early transgressive, condensed deposits resting over the erosional ravinement surface. Four bivalve shells have been sampled from this large slab of indurated shell-rich sand for AMS-^14^C dating (Fig. 5, Supplementary Material Table S1). The oldest ^14^C calibrated age refers to the brackish-lagoonal bivalve *Cerastoderma glaucum* (9,214 ± 244 yr BP) whilst a shallow-marine bivalve *Chamelea gallina* yielded an age of 8,496 ± 309 yr BP. Younger ages have been obtained from two shells, i.e. *Loripes lucinalis* and *Flexopecten glaber*, that provided calibrated ages of 7,349 ± 200 and 7,862 ± 233 yr BP, respectively.

## Discussion

The North Adriatic bio-concretionned rocky buildups have defied for long a convincing explanation of their genetic processes. Their nature could not be unraveled by a sole ecological approach given that epifaunal and algal component is mainly a later exploitation of a pre-existing lithified substrate. Current views favor hydrocarbon-enriched fluid seepage through porous sediment as the leading process driving to early lithification of such type of rocky buildups on the North Adriatic continental shelf e.g. refs 14–16. The present study reinforces and complements this hypothesis by disclosing the role of non-hydrocarbon fluid seepage in the genesis of these peculiar habitats.

Previous studies have documented that the architecture of the subsoil off the Venice lagoon is extremely similar to that revealed by high-resolution seismic profiles of the study area^4, 18^. According to these reconstructions, the lower unit is interpreted as the Late Pleistocene aggrading alluvial plain (Fig. 3, Supplementary Material Figures S2 and S3), crossed by fluvial channels, whereas the upper unit is the distal part of the Holocene coastal wedge that downlaps the exposed top of the drowned floodplain (Fig. 3, Supplementary Material Figures S2 and S3). At the seismic scale, the bio-concretionned rocky buildups grew on top of the late Pleistocene alluvial plain, and in particular above buried channels (Fig. 3, Supplementary Material Figure S3).

**Figure 6:**
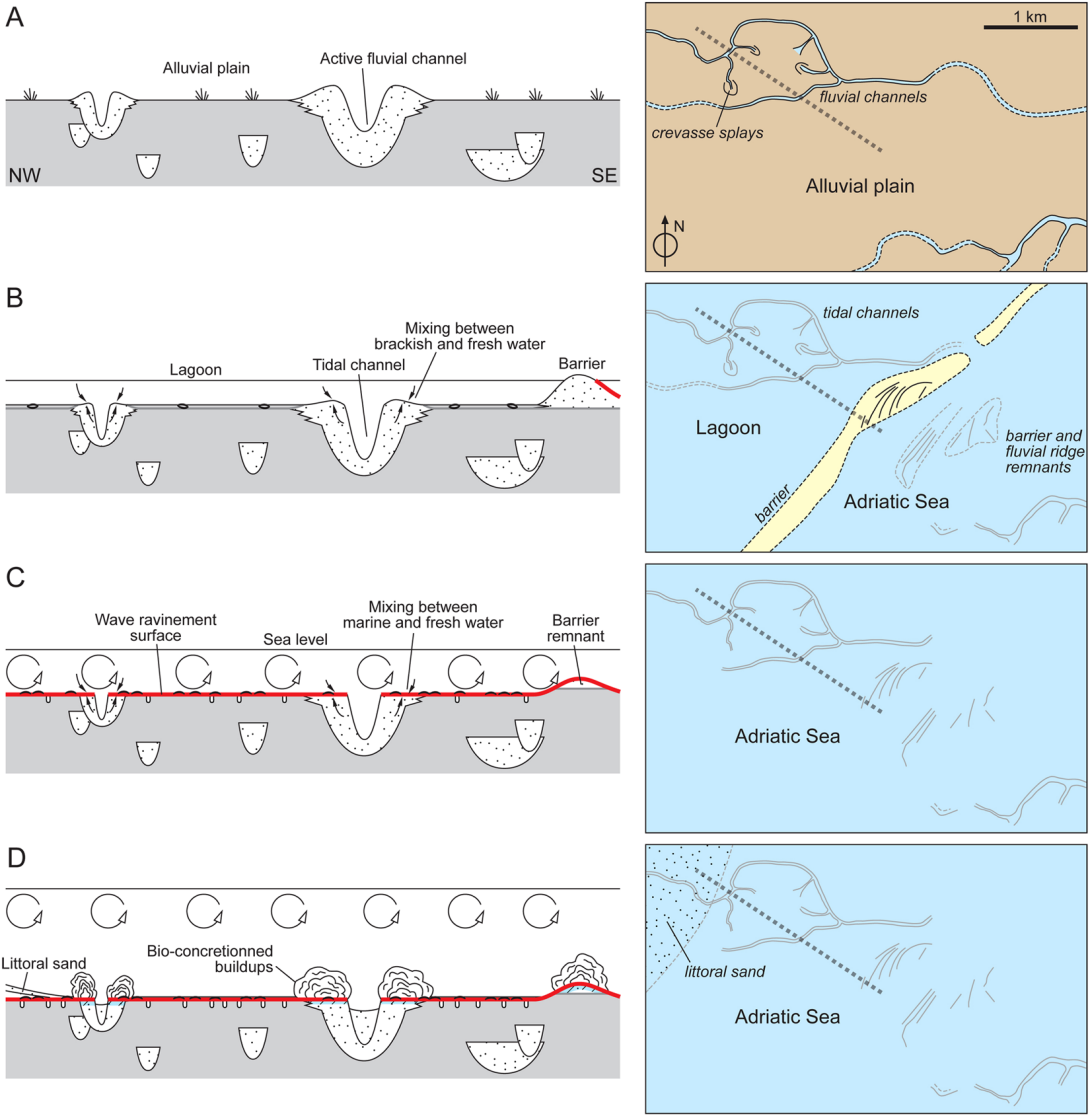
Genetic model of coralligenous buildups within the geological evolution framework of the Venice offshore. The trace of the area covered by the sketch is provided in Fig. 1, red rectangle. Dashed grey lines show the position of the profiles. (**A**) Fluvial ridges developed on the LGM alluvial plain. (**B**) The study area was temporarily transformed in a lagoon protected seaward by a barrier island during the post-LGM transgression. (**C**) The lagoon was rapidly overstepped due to the eustatic sea-level rise and wave action produced a wave-ravinement surface. (**D**) The cementation of the previously deposited sandy sediments, induced by the mixing between marine and fresh water, led to formation of stable substrates that favored the growth of bio-concretionned buildups.

It is inferred that the meandering and winding features found on the Adriatic seafloor originated as fluvial ridges aggrading on the Last Glacial Maximum (LGM) alluvial plain (Fig. 6A). During major floods, the fluvial levees were locally breached and crevasse splays accumulated on the floodplain (Fig. 6A). During the post-LGM transgression, the study area was temporarily transformed in a lagoon protected seaward by a barrier island, and the rivers were probably converted in tidal channels (Fig. 6B). The observed hectometer-scale arc-shaped features found in the central part of the study area (Fig. 2A) are inferred to represent the remnants of beach ridges forming the barrier island (Fig. 6B). The lagoon was rapidly overstepped due to the eustatic sea-level rise and wave action reworked the barrier, the lagoonal deposits, the previous surface of subaerial exposure and the fluvial ridges, producing a wave-ravinement surface (WRS)^19^ that coincides with the boundary between the two units found in the seismic profiles (Figs 3A,B and 6C, Supplementary Material Figures S2 and S3). The reworking of deposits accumulated in different sedimentary environments is confirmed by the presence of a mixed fauna with freshwater (*Lymnaea*, Planorbiidae), brackish-lagoonal (*Cerastoderma glaucum*, *Loripes lucinalis*) and shallow-marine (*Chamelea gallina*, *Flexopecten glaber*) taxa in the shell lag that mantles the WRS, which corresponds to the cemented shell-rich layer found at the seafloor (Fig. 4C). The lag, accumulated between 9 and 7 cal. ka BP (Supplementary Material Figure S5B), testifies a phase of condensed sedimentation during the late post-LGM eustatic sea-level rise.

The overall lack or very modest thickness of Holocene sediments offshore the Holocene coastal wedge suggests persisting conditions of scarce sediment supply after the transgressive phase. These conditions have probably favored the early cementation of the sandy sediments pertaining to the Late Pleistocene channel levees and the remnants of the barrier islands exposed on the seafloor. The analysis of the cements confirmed that this process took place under marine conditions, although the typology of the cement suggests the interaction between marine water and fluids related to onshore freshwater, likely discharged at sea by a sealed water-table. Whether meniscus cements are normally accounted as products of carbonate precipitation within the meteoric/marine vadose zone e.g. refs 20–22, isopachous rims of calcite are rather typical of precipitation within the phreatic zone^22, 23^. The investigated samples of *tegnùe* clearly document a marine phreatic paragenesis characterized by multiple generations of scalenohedral and microcrystalline calcite (Fig. 4D, Supplementary Material Figure S5C,D). Processes of mixing of marine and meteoric water at the phreatic mixing zone coupled with the CO_2_ outgassing of interstitial mixed meteoric/marine water have probably induced the initial dissolution of the phreatic cements e.g. refs 21–24 followed by the precipitation of a second generation of cement (Fig. 4D).

The presence of fresh submarine groundwater as well as the occurrence of submarine groundwater discharges are quite common in the intertidal zone and up to a few km offshore, depending on the stratigraphic setting of the shallow aquifer system [e.g. ref. 25 and reference therein]. At the time of the early cementation of the sandstones the area of the Chioggia’s *tegnùe* was just shifted from lagoon/intertidal to shallow sea following the post-LGM transgression. In that scenario the shallow aquifers were the sandy paleochannel fill systems crossing the upper Pleistocene alluvial plain e.g. ref. 26. The presence of low salinity groundwater beneath the condensed Holocene deposits is therefore a plausible hypothesis.

Based on the available ^14^C dating, the lagoon-barrier system in the study area of Chioggia developed at ca. 9 cal. ka BP and it was probably contemporaneous of a coastal wedge that developed off the modern Tagliamento delta (NE of the study area), which was the precursor of a cemented sand ridge, referred to as the “*Trezza Grande*”^27, 28^. A third coeval coastal wedge developed between the study area and the “*Trezza Grande*”; this system has been subsequently reworked and transformed in a field of sand ridges found at a depth of 20–24 m^19^ corresponding to the depth of the ancient lagoon-barrier system recognized in the present study. The contemporaneous development of such coastal systems may have been favored by a phase of slow relative sea-level rise, between 9 and 8 cal. ka BP, probably correlated with the well-known 8,200 year cold event^29^. The coastal systems were probably drowned between 8 and 7 cal. ka BP, after the cold event, as demonstrated by the mixing between lagoonal and shallow-marine faunas in the recognized transgressive lag, representing a time interval spanning between 9 and 7 cal. ka BP. The lithification occurred soon after this early step of the transgression since cementing only very shallow-marine taxa and is, therefore, set at about 7 cal. ka BP.

After lithification, the channels acted as sediment traps allowing protection from waves and currents that prevent the accumulation on the flat shelf areas. The cemented sediments acted as stable substrates that favored the growth of the coralligenous buildups (Figs 1B and 2C), which retrace the trend of the ancient fluvial systems and beach ridges (Figs 2B and 6D).

Generally, this type of bioconstructions grows on stable substrates and has an optimal depth ranging between 30 and 60 m in the modern Mediterranean Sea^30–32^. Coralligenous buildups, showing a thickness similar to those studied, are common in late Quaternary marine terraces in southern Italy, and consist of reefs with patchy distribution or more extensive bioconstructions grown on transgressive lags and well-cemented, condensed shell beds^33–38^. Nevertheless, the occurrence of coralligenous buildups developed above older fluvial meandering systems is an unusual feature never observed before in the Mediterranean Sea.

## Methods

High resolution seismic data were collected by CHIRP and BOOMER sub-bottom profiles totaling about 140 line-km (Supplementary Material Figure S1) by the R/V URANIA and LITUS. Swath bathymetry and Side Scan Sonar surveys were acquired on board the R/V ASTREA^39^ and made available by ‘Regione Veneto’.

The scientific underwater SCUBA diver teams performed more than 200 dives for geomorphological reconnaissance (Supplementary Material Movie 1) and sediment sampling (Supplementary Material Movies 2–3) by push-cores handling in soft and unconsolidated sediments; Supplementary Material Figure S6A) and by handling hammer (Supplementary Material Figure S6B) and hydraulic underwater hammer drill in cemented sands and bio-concretionned layers (Supplementary Material Figures S6C–F).

Well preserved and whole shells extracted from slab MT_01_14 were analysed for AMS-^14^C dating at the Beta Analytic laboratory (Miami, USA). Conventional radiocarbon ages were calibrated using the Calib7.1 program^40^ and the calibration curve Marine13.14c^41^ (Supplementary Material Table S1). A Delta-R value of 46 ± 104 was used for the calibration. The petrographic and microfacies analyses were performed on uncovered thin sections (30 μm thickness) at ISMAR-CNR (Bologna). Chemical elemental composition was obtained using a CamScan MX 3000S scanning electron microscope (SEM) with a LaB6 cathode, equipped with a system for backscattered electrons imaging (BEI) and energy-dispersive X-ray spectroscopy (EDS) at University of Padua. SEM-EDS investigations (operating conditions: ca. 20 kV accelerating voltage) were performed on carbon coated freshly broken samples and thin sections. Samples of bulk rock were powdered for mineralogical analyses and analyzed using a Philips PW 1480, CuK1, X-ray diffractometer. The resulting spectra were compared with reference XRD spectra using the Crystal Sleuth software^42^.

## Electronic supplementary material

Supplementary Material

Supplementary Movie 1: Overview of the bio-concretionned rocky buildups

Supplementary Movie 2: Sediment and rock sampling

Supplementary Movie 3: Drilling
