# Supplementary material for: Paleochannel and beach-bar palimpsest topography as initial substrate for coralligenous buildups offshore Venice, Italy
Source: Sci Rep. 2017 May 2;7:1321. doi: 10.1038/s41598-017-01483-z (PMC5430969; doi:10.1038/s41598-017-01483-z)
Supplement: Supplementary file 1 — Supplementary Material [file 41598_2017_1483_MOESM1_ESM.pdf]

Supplementary Material

for

"Paleochannels and beach-bar palimpsest topography as initial substrate for coralligenous buildups offshore Venice, Italy"

Luigi Tosi[1]*, Massimo Zecchin[2], Fulvio Franchi[3,4], Andrea Bergamasco[1], Cristina Da Lio[1], Luca Baradello[2], Claudio Mazzoli[5], Paolo Montagna[4,6], Marco Taviani[4,7,8], Davide Tagliapietra[1], Eleonora Carol[9], Gianluca Franceschini[10], Otello Giovanardi[10], Sandra Donnici[1]

[1] Institute of Marine Sciences, National Research Council, Arsenale - Tesa 104, Castello 2737/F, 30122 Venezia, Italy.

[2] National Institute of Oceanography and Experimental Geophysics, Borgo Grotta Gigante, 42/c, 34010 Sgonico, Trieste, Italy.

[3] Botswana International University of Science and Technology, Private Bag 16, Plot 10071 Palapye, Botswana.

[4] Institute of Marine Sciences, National Research Council, Via Gobetti 101, 40129 Bologna, Italy.

[5.] Department of Geosciences, University of Padova, Via Gradenigo 6, 35131 Padova, Italy.

[6] Laboratoire des Sciences du Climat et de l'Environnement LSCE/IPSL, CEACNRS-UVSQ, Université Paris-Saclay, Avenue de la Terrasse, Gif-sur-Yvette, Île-de-France, 91198, France.

[7] Biology Department, Woods Hole Oceanographic Institution, 266 Woods Hole Rd, Woods Hole, Ma. 02543, USA.

[8] Stazione Zoologica Anton Dohrn, Villa Comunale, 80121 Napoli, Italy.

[9] Centro de Investigaciones Geologicas, Consejo Nacional de Investigaciones Científicas y Tecnicas, Diagonal 113 N275, B1904DPK, La Plata, Argentina.

[10] Italian National Institute for Environmental Protection and Research, Loc. Brondolo, 30015 Chioggia, Venezia, Italy.

Correspondence and requests for materials:
L.T., luigi.tosi@ismar.cnr.it
A.B., andrea.bergamasco@ismar.cnr.it

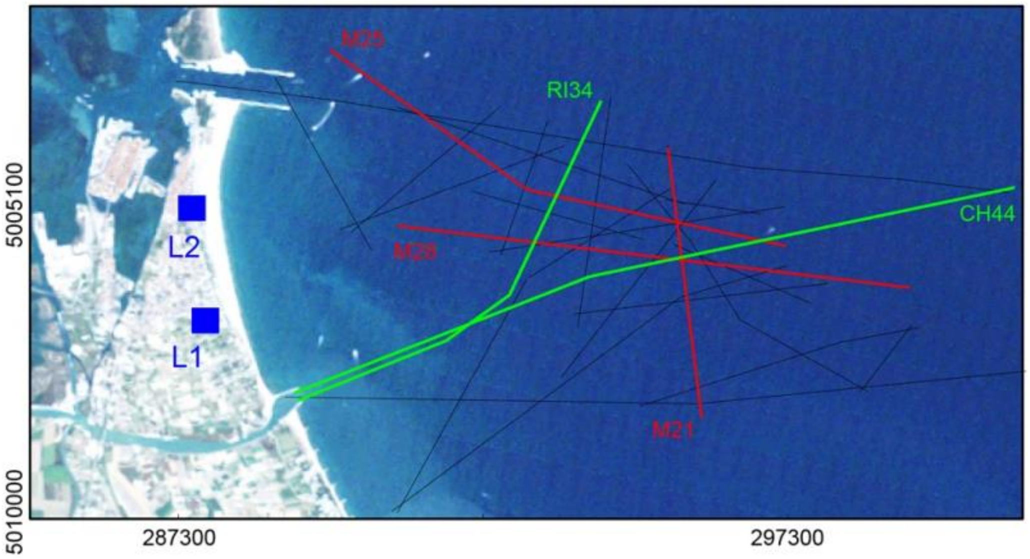

Figure S1. Position of the seismic lines (UTM33N WGS84). The base map used is a Landsat image obtained from the US Geological Survey – Earth Resources Observation and Science (EROS) Center and composed in ESRI ArcMAP 9.3.

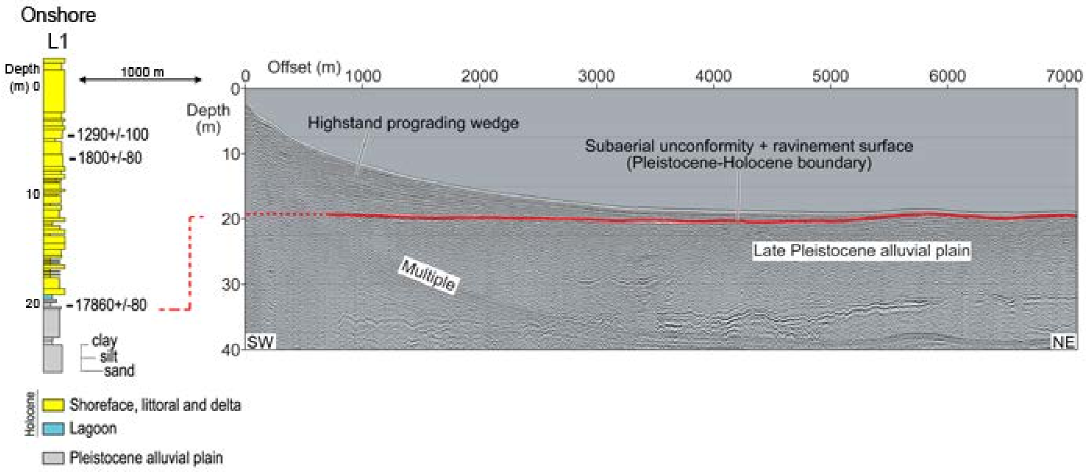

Figure S2. Seismic line RI34. Details of paleoenvironments, stratigraphy, and chronology of core L1 is provided by Tosi et al., 1994; Tosi et al., 2007a; Tosi et al. 2009. Seismic line and core positions are shown in S1.

Tosi, L., Rizzetto, F., Bonardi, M., Donnici, S., Serandrei Barbero, R. & Toffoletto, F. Note illustrative della Carta Geologica d'Italia alla scala 1: 50.000, Foglio 148–149 Chioggia-Malamocco, APAT, Dip. Difesa del Suolo, Servizio Geologico d'Italia, SystemCart, Roma, pp. 164, 2 Maps (2007a).

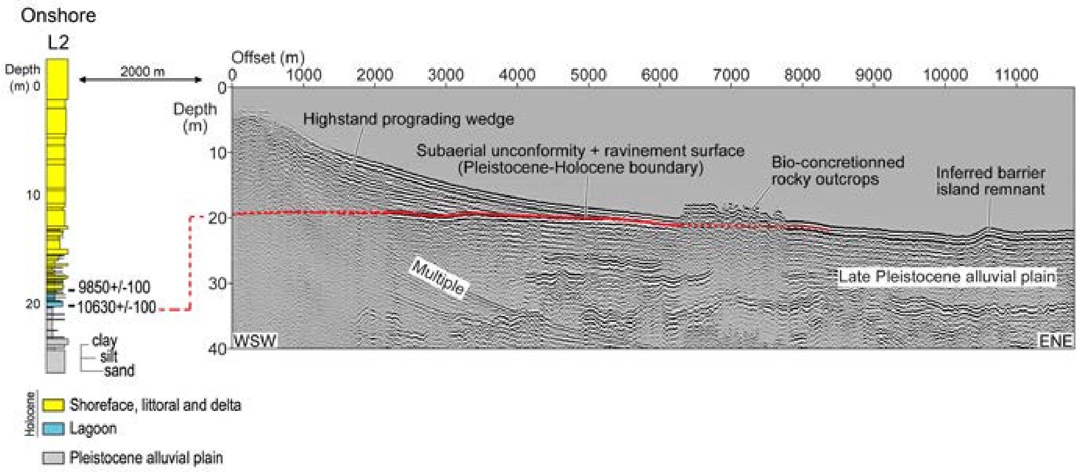

Figure S3. Seismic line CH44. Details of paleoenvironments, stratigraphy, and chronology of core L2 [Tosi et al., 2007a]; Seismic line and core positions are shown in S1.

Tosi, L., Rizzetto, F., Bonardi, M., Donnici, S., Serandrei Barbero, R. & Toffoletto, F. Note illustrative della Carta Geologica d'Italia alla scala 1: 50.000, Foglio 148–149 Chioggia-Malamocco, APAT, Dip. Difesa del Suolo, Servizio Geologico d'Italia, SystemCart, Roma, pp. 164, 2 Maps (2007a).

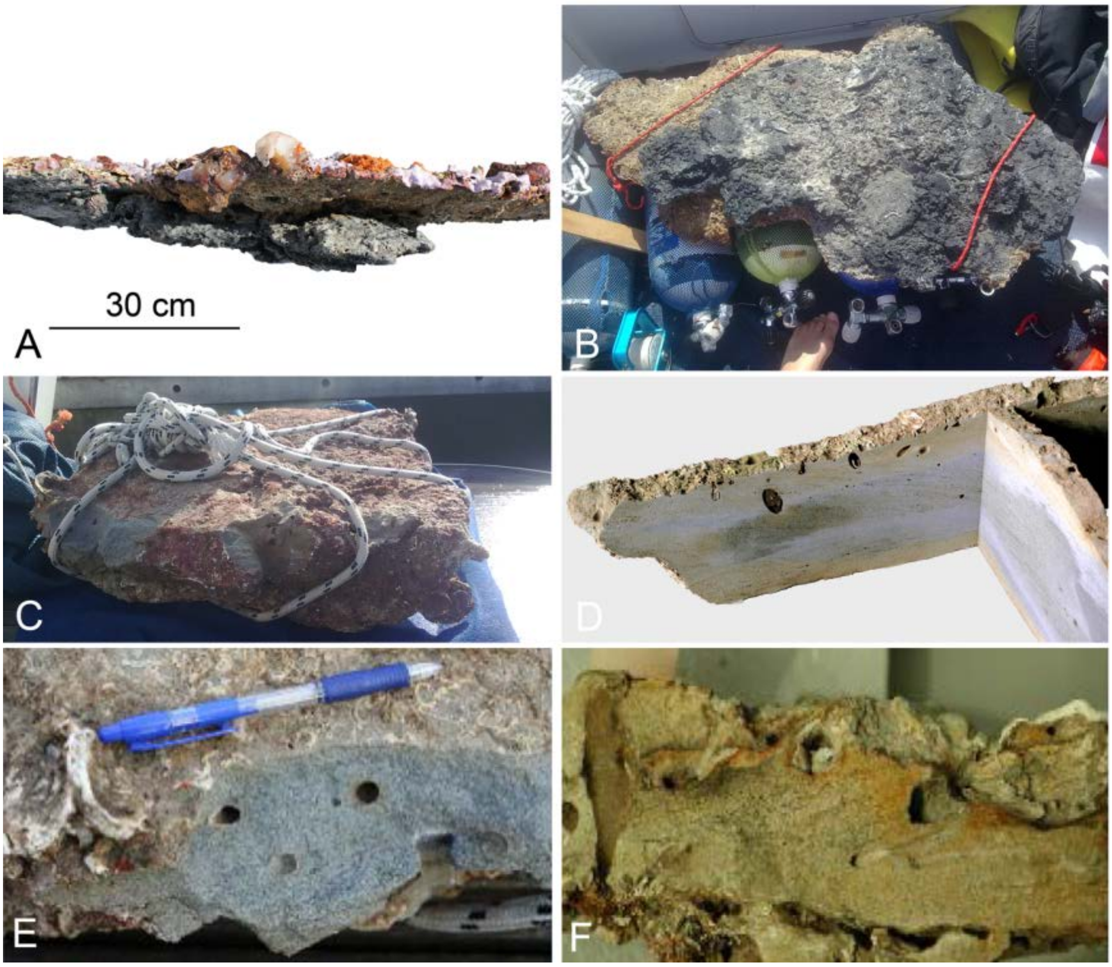

Figure S4. Samples of cemented sand layers. A) vertical and B) bottom views of the shell-rich coarse layer of Fig. 2E. C) Bulk, D) orthogonal slices and E) slab of the cemented sample shown in Fig. 2F. F) cemented sand layers with bioconstructions.

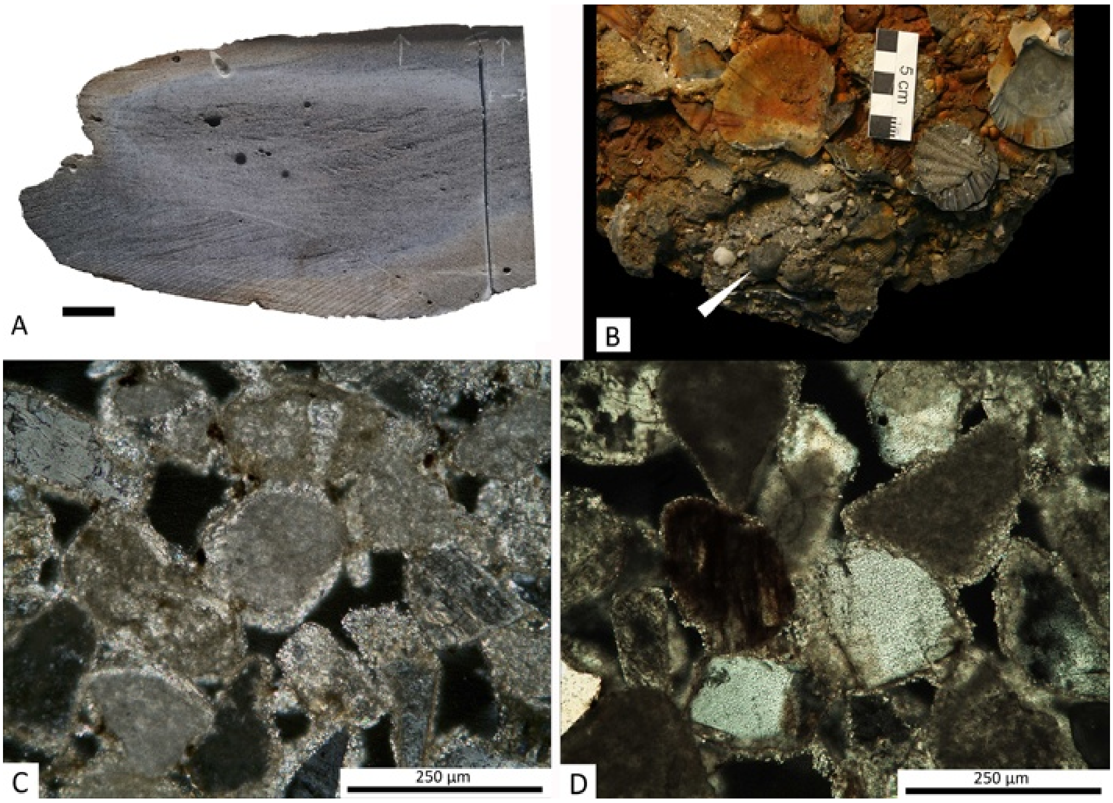

Figure S5. A) Sample of cross stratified sand of Fig. 2F. The foresets are particularly clear in the centre of the sample. B) Shell-rich cemented sand showing abundant biosomes. C-D) Isopachous rims of scalenohedral calcite cementing mixed carbonate siliciclastic sand. Cross polarized light.

Sample	Species	Weight (mg)	$\delta^{13}C$ (‰)	Conventional Radiocarbon Age (yrs BP)	Percent Modern Carbon (pMC)	Calibrated Age (yrs BP)*	2σ range (yrs BP)
TE-1	*Chamelea gallina*	67.53	-0.9	8070 ± 30	36.6 ± 0.1	8496 ± 309	8266 - 8805
TE-2	*Loripes lucinalis*	28.14	-0.3	6880 ± 30	42.5 ± 0.2	7349 ± 200	7153 - 7549
TE-3	*Flexopecten glaber*	27.65	-1.1	7450 ± 30	39.6 ± 0.2	7862 ± 233	7646 - 8095
TE-4	*Cerastoderma glaucum*	35.14	-4.3	8620 ± 30	34.2 ± 0.1	9214 ± 244	8974 - 9458

Table S1. Conventional and calibrated ages* from sample MT_01_14.

* Reimer, P., Bard, E., Bayliss, A., Beck, J.W., Blackwell, P.G., Bronk Ramsey, C., Buck, C.E., Cheng, H., Edwards, R.L., Friedrich, M., Grootes, P.M., Guilderson, T.P., Haflidason, H., Hajdas, I., Hatt, Ã., C., Heaton, T.J., Hogg, A.G., Hughen, K.A., Kaiser, K.F., Kromer, B., Manning, S.W., Niu, M., Reimer, R.W., Richards, D.A., Scott, E.M., Southon, J.R., Turney, C.S.M. & van der Plicht, J. IntCal13 MARINE13 radiocarbon age calibration curves 0-50,000 years calBP. Radiocarbon **55**, 1869-1887 (2013).

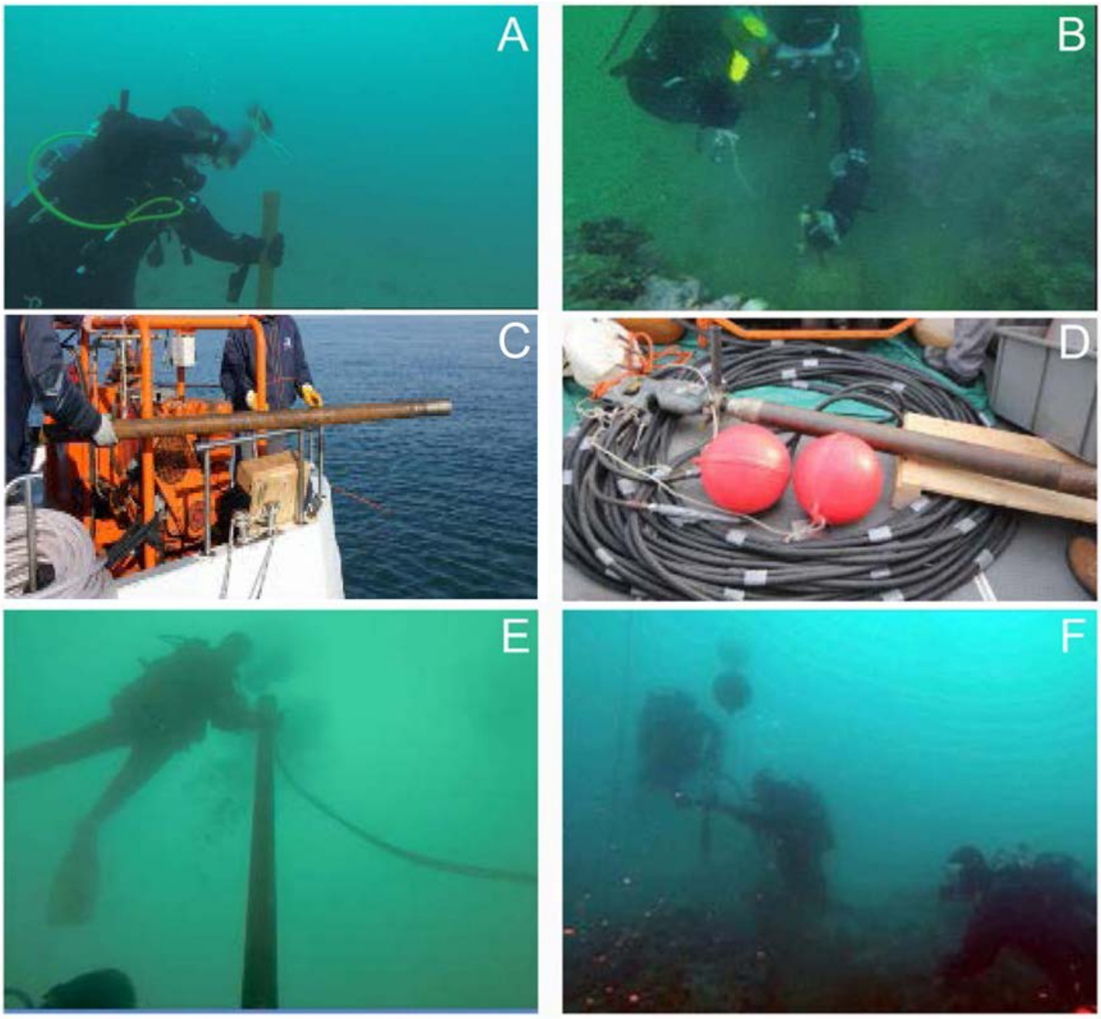

Figure S6. Samplings: A) Push-cores handling in soft and unconsolidated sediments; B) Handling hammer; C-F) Hydraulic underwater hammer drill in cemented sands and bio-concretioned layer.

Supplementary Movie 1: Overview of the bio-concretionned rocky buildups.

Overview of the bio-concretionned rocky buildups, 5-8 km offshore Chioggia, South of Venice. Recognizing their origin has been always hampered by the difficulty of getting samples of diagnostic value, especially for the poor visibility conditions encountered by divers during the visual surveys due to the high water turbidity.

Supplementary Movie 2: Sediment and rock sampling.

The scientific underwater SCUBA diver teams performed more than 200 dives for geomorphological reconnaissance and sediment sampling by push-cores handling in soft/unconsolidated sediments and by handling hammer in cemented sands and bio-concretionned layers.

Supplementary Movie 3: Drilling.

Rock sampling by hydraulic underwater hammer drill in cemented sands and bio-concretionned layers.
